# Ubiquitous healthy diatoms in the deep sea confirm deep carbon injection by the biological pump

**DOI:** 10.1038/ncomms8608

**Published:** 2015-07-09

**Authors:** S. Agusti, J. I. González-Gordillo, D. Vaqué, M. Estrada, M. I. Cerezo, G. Salazar, J. M. Gasol, C. M. Duarte

**Affiliations:** 1 Red Sea Research Center, King Abdullah University of Science and Technology (KAUST), Thuwal 23955-6900, Kingdom of Saudi Arabia; 2 Department of Global Change Research, IMEDEA (CSIC-UIB), Miquel Marqués 21, Esporles 07190, Spain; 3 Department of Biology, Campus de Excelencia Internacional del Mar (CEIMAR), Universidad de Cádiz, Puerto Real (Cádiz) 11510, Spain; 4 Institut de Ciències del Mar, CSIC, Passeig Marítim de la Barceloneta 37-49, Barcelona, Catalunya E 08003, Spain

## Abstract

The role of the ocean as a sink for CO_2_ is partially dependent on the downward transport of phytoplankton cells packaged within fast-sinking particles. However, whether such fast-sinking mechanisms deliver fresh organic carbon down to the deep bathypelagic sea and whether this mechanism is prevalent across the ocean requires confirmation. Here we report the ubiquitous presence of healthy photosynthetic cells, dominated by diatoms, down to 4,000 m in the deep dark ocean. Decay experiments with surface phytoplankton suggested that the large proportion (18%) of healthy photosynthetic cells observed, on average, in the dark ocean, requires transport times from a few days to a few weeks, corresponding to sinking rates (124–732 m d^−1^) comparable to those of fast-sinking aggregates and faecal pellets. These results confirm the expectation that fast-sinking mechanisms inject fresh organic carbon into the deep sea and that this is a prevalent process operating across the global oligotrophic ocean.

## Abstract (web summary)

Sinking of organic matter represents the dominant mechanism for sequestration of anthropogenic CO_2_ in the deep sea. Here, the authors report the presence of healthy photosynthetic cells in the deep dark ocean, implying the fast injection of fresh organic carbon at depth across the global oligotrophic ocean.

## Graphical abstract

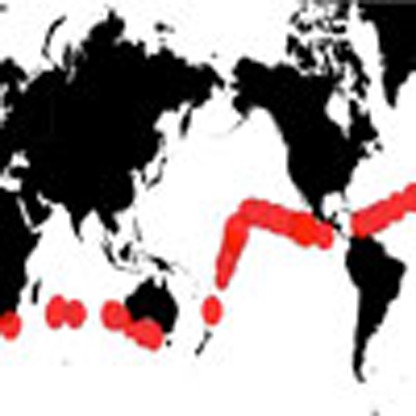

The role of the ocean as a sink for anthropogenic CO_2_ is critically dependent on the transport of carbon to depths below 1,000 m, where it is removed from ventilating back to the atmosphere over centennial timescales^1^,^2^. Ocean plankton contributes to remove CO_2_ through sinking of particles, transporting organic carbon at depth, the so-called biological pump^3^,^4^. Sinking rates of individual phytoplankton cells are very slow, at ∼1.5 m d^−1^ (ref. 5) expected for diatom cells^6^. However, Smayda^7^ suggested that a range of physical and biological (aggregation, downwelling and density inversion currents, packaging of cells in faecal pellets) mechanisms can accelerate phytoplankton-sinking rates *in situ*^8^,^9^ beyond the rates expected for single cells. Subsequent laboratory experiments and observations, mostly derived from the epi- and mesopelagic layers of productive regions of the ocean, have documented phytoplankton cells within aggregates and faecal pellets to sink at rates of 10–1,000 m d^−1^ (refs 9, 10, 11, 12). These fast-sinking rates are consistent with estimates of sinking rates derived using geochemical tracers, such as ^230, 234^Th isotopes^13^,^14^, reports of events of accelerated phytoplankton export^15^,^16^, and calculations suggesting high carbon use by heterotrophs in the deep ocean^17^,^18^. Hence, that the transport of organic carbon at depth can be greatly accelerated by processes involving the packaging of phytoplankton cells within aggregates and faecal pellets is now well established^19^,^20^,^21^. However, whether these mechanisms delivering fresh organic carbon reach the deep sea and how prevalent they are across the oligotrophic regions of the ocean remains to be tested.

Research since the 1960–1970s reported the occasional presence of well-preserved phytoplankton cells in the deep sea^22^,^23^,^24^,^25^; however, these observations, which could signal at rapid sinking rates^7^, were considered anecdotal. Using new developments we tested the presence of healthy phytoplankton cells in the deep sea (2,000–4,000 m depth) along the Malaspina 2010 Circumnavigation Expedition, a global expedition sampling the bathypelagic Atlantic, Indian and Pacific Oceans^26^,^27^,^28^. In particular, we used a new microplankton sampling device, the Bottle–Net (Supplementary Fig. 1), 16S rDNA sequences, flow cytometric counts, vital stains and experiments to explore the abundance and health status of photosynthetic plankton cells in bathypelagic waters between 2,000 and 4,000 m depth along the circumnavigation track (Fig. 1).

## Results

### Phytoplankton cells were ubiquitous in the deep sea

**Figure 1:**
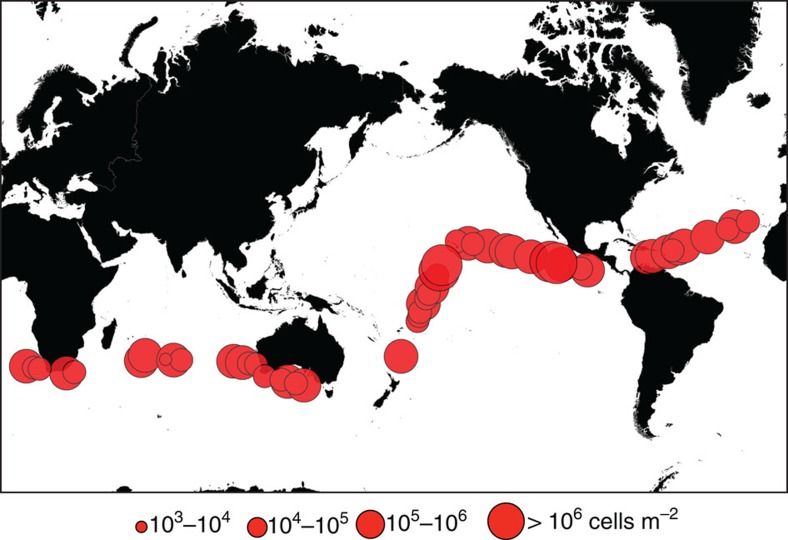
Abundance of phytoplankton cells in the deep ocean. Microphytoplankton (>20 μm diameter) were collected between 2,000 and 4,000 m depth using the Bottle-Net across the subtropical, and tropical areas of the Atlantic, Indian and Pacific Oceans sampled during the Malaspina 2010 Circumnavigation Expedition. The diameter of the symbols is scaled to the integrated (2,000–4,000 m) bathypelagic microphytoplankton cell abundance.

**Figure 2:**
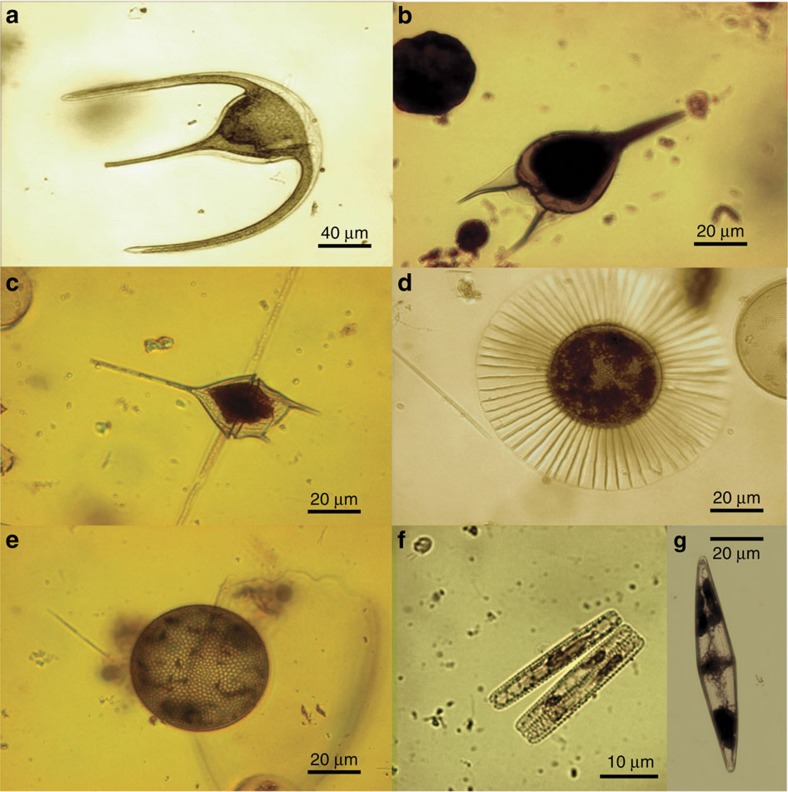
Intact phytoplankton cells in the dark deep ocean. Phytoplankton cells collected by the Bottle-Net between 2,000 and 4,000 m observed under the optical microscope included dinoflagellate (**a**–**c**) and diatom (**d**–**g**) genera. Most phytoplanktonic cells corresponded to vegetative cells.

The samples retrieved in the Malaspina 2010 Circumnavigation Expedition revealed the ubiquitous presence in the deep, bathypelagic dark ocean of morphologically well-preserved (photosynthetic) microphytoplankton cells, characteristic of the lighted layers of the surface ocean (Fig. 2). The highest concentrations of phytoplankton in the deep sea were found in the Equatorial Pacific. The concentration of phytoplankton cells in the deep ocean was low, averaging 2.5 × 10^5^ cells m^−2^ in the 4,000–2,000 m depth layer (range<50 to >500 cells m^−3^, Fig. 1, Table 1). However, phytoplankton cells were more abundant than heterotrophic ciliates (Table 1, Supplementary Fig. 1), the organisms hitherto believed to dominate deep sea microplankton communities^29^, and the ratio of photosynthetic to heterotrophic protist cells did not differ between surface and deep waters (Table 1).

**Figure 3:**
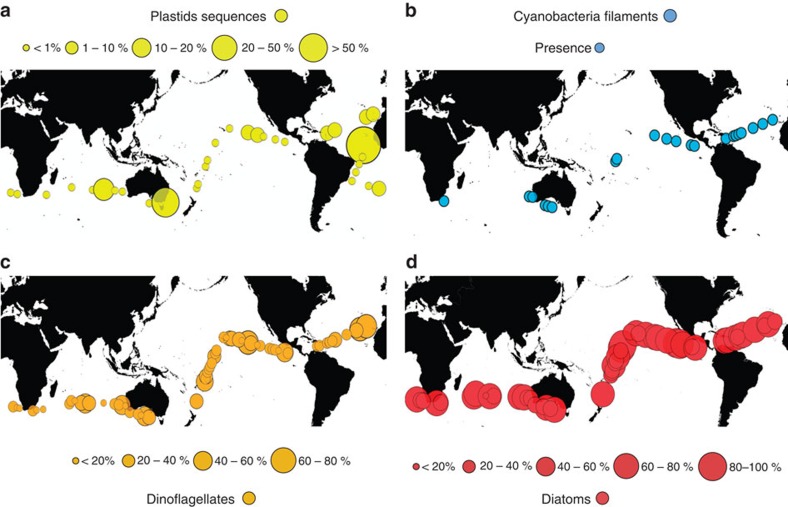
Phytoplankton community in the bathypelagic ocean. (**a**) The per cent of 16S rDNA sequences of 4,000 m communities comprised photosynthetic cells (as the % algal sequences (that is, cyanobacteria and algal chloroplast) relative to the total sequences registered). The dots indicated the stations where DNA was analysed and the diameter is scaled to the % of algal+cyanobacteria sequences relative to the total sequences registered as indicated in the scale in the top. (**b**) Stations where cyanobacteria filaments (*Trichodesmium* spp. and *Richelia* sp.) were observed in the deep ocean (2,000–4,000 m). (**c**) Percentage of the total phytoplankton cells corresponding to Dinoflagellates in the deep ocean (2,000–4,000 m). (**d**) Percentage of the total phytoplankton cells corresponding to Diatoms, dominated by centric forms, in the deep ocean (2,000–4,000 m). The diameter of the symbols in **c**,**d** are scaled to the % of cells in the photosynthetic community, as indicated in the scale at the bottom of the graphs.

Microphytoplankton communities in the deep sea were dominated by diatoms (81.5%), while photosynthetic dinoflagellates dominated the community in the Eastern Subtropical Atlantic (Fig. 3). Indeed, diatoms exerted a greater dominance in the deep-sea microphytoplankton community than in surface waters (Table 1). Nitrogen-fixing cyanobacteria were occasionally abundant, including *Trichodesmium* sp. trichomes in the deep waters of the Subtropical Atlantic and the Equatorial Pacific, and symbiotic N_2_-fixing *Richelia* sp. within large *Rhizosolenia* (diatom) hosts in the Eastern Indian Ocean (Fig. 3; Table 1). Aggregates containing *Synechococcus* cells and other phytoplankton were also observed in deep waters. Single picophytoplankton cells were also detected using flow cytometry in the samples (Table 1; Supplementary Fig. 2), supporting previous reports^25^. Further, we also consistently observed the presence of significant numbers of 16S rDNA sequences affiliated to algae plastids and to cyanobacteria in samples collected at 4,000 m depth, where eukaryotic algae and cyanobacteria comprised up to 68.9% of the sequences in some microbial communities (Fig. 3).

Three observations pointed at the overlying waters, rather than lateral transport, as the source of the phytoplankton cells found in the deep sea: (1) the general correspondence between the community structure of microalgae in the surface and corresponding deep-sea samples across stations (Supplementary Table 1); (2) the occurrence in both surface waters and the corresponding bathypelagic waters of relatively rare taxa, as illustrated by the large *Rhizosolenia* diatom cells containing the symbiotic N_2_-fixing cyanobacteria *Richelia* encountered in both epi- and bathypelagic waters of the Indian Ocean near Western Australia (Supplementary Table 1); and (3) that resting stages, which could be transported laterally within the deep sea, were not detected in the deep-sea microphytoplankton community for those taxa for which morphologically distinct resting stages have been reported, including dinoflagellates, and the diatom genera *Chaetoceros*, *Leptocylindrus* and *Rhizosolenia*^30^.

### Alive phytoplankton cells in the bathypelagic ocean

**Figure 4:**
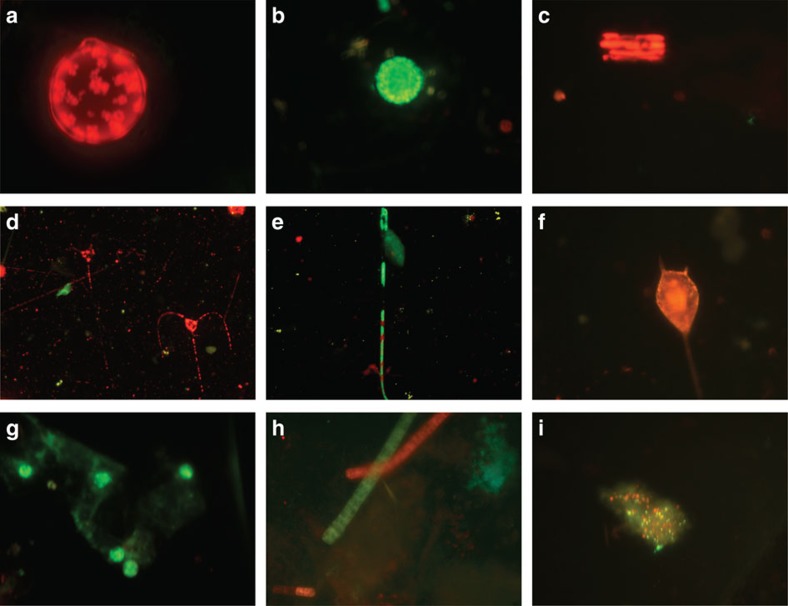
Testing the cell membrane permeability of bathypelagic phytoplankton. After stained with the double vital stain Bac-light Kit the living phytoplankton cells collected by the Bottle-Net fluoresce green and the dead cells fluoresce red when observed under the epifluorescence microscope. (**a**) Dead centric diatom; (**b**) alive centric diatom; (**c**) dead pennate diatom cells in a colony; (**d**) dead cells of the dinoflagellate *Ceratium sp.*; (**e**) alive cell of the dinoflagellate *Ceratium fusus*; (**f**) dead cell of *Ceratium sp.* red; (**g**) a group of alive cells aggregated in a particle; (**h**) dead (red) and alive (green) trichomes of the cyanobacteria *Trichodesmium*; (**i**) an aggregate of cells from the deep ocean containing picophytoplankton and bacteria. As this figure is to show differences in fluorescence colour there are no scale bars; the organisms and cells shown are all on the order of micrometres.

**Figure 5:**
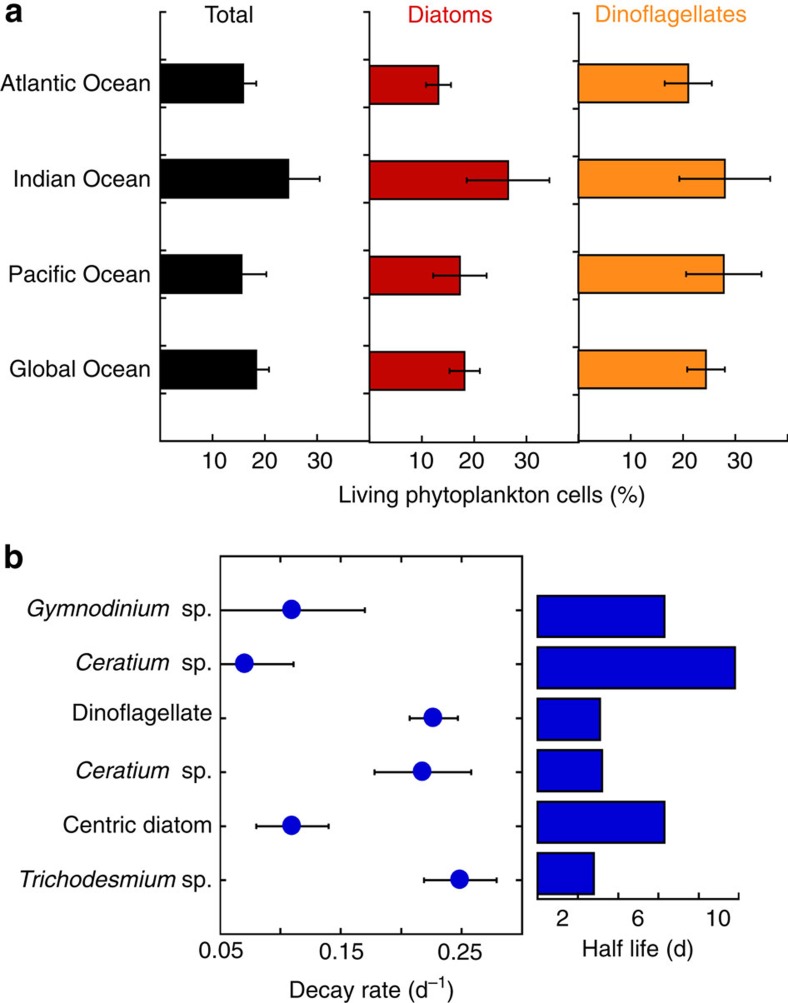
Living phytoplankton cells (>20 μm diameter) in the bathypelagic ocean and cell survival in the dark ocean. (**a**) The percentage (mean±s.e.) of living microphytoplankton cells found in the deep water column (2,000–4,000 m) of the Atlantic, Indian and Pacific Oceans, and for the global deep ocean sampled in the Malaspina 2010 Circumnavigation Expedition. Black columns represent the mean percentages of living cells for the total microphytoplankton cells encountered (±s.e.), and red and orange columns represent the mean percentages of living cells within diatom and dinoflagellate communities, respectively. Living and dead cells were identified by using a double vital stain to test cell membrane permeability. (**b**) Experimentally derived mean (±s.e.) phytoplankton living cell's decay rates (d^−1^) and half-lives of alive cells (days) for populations sampled at the photic surface layer and incubated in 4,000 m depth water and under deep ocean conditions (dark and cold temperature) for more than a month. Centric diatom: undetermined centric diatom. Dinoflagellate: undetermined dinoflagellate.

A proportion, 18.4±2.4% on average (Figs 4 and 5), of the phytoplankton cells present in the deep ocean had intact plasma membranes^31^,^32^, thereby demonstrating the presence of living phytoplankton cells in the deep ocean (Fig. 4). Experimental assessments of cell mortality of photic layer phytoplankton under the conditions of the dark deep ocean revealed high decay rates of living populations, with half-lives of living cells ranging from 3 to 10 days (Fig. 5; Supplementary Fig. 3). The time for living cells to decline to 18% of the community, the average cell viability in populations of photosynthetic microplankton retrieved from the deep ocean (Fig. 5), ranged between 6.8 and 24.2 days.

## Discussion

Microphytoplankton sinking as single cells at rates in the order of 1.5 m d^−1^ (refs 5, 6) would take 3.8–7.3 years to reach 2,000–4,000 m depth, respectively. Previous experiments demonstrated that phytoplankton populations may be preserved in the dark for over 2 months^33^; however, these experiments did not examine the rate of decay of viable cells in the dark. Our experimental results, which did not examine preservation but survival rates, show much shorter lifespans of phytoplankton cells in the conditions of the dark ocean. Our experiments imply that most cells of the phytoplankton communities we sampled in the deep ocean would be dead after 1 month and no living cells would be expected to remain in the population after transit times in excess of a year. Moreover, even most dead microphytoplankton cells in our bathypelagic samples were also morphologically well preserved, which would not be possible if they had left the photic layer months to years ago. Hence, the presence of healthy microphytoplankton cells between 2,000 and 4,000 m depth can only be explained by very fast-sinking rates.

On the basis of our experimental results we calculate that for 18% of phytoplankton cells to be still alive, populations sinking below the photic layer must take 6.8–24.2 days, at sinking rates of 124–435 m d^−1^, to reach 3,000 m depth (midpoint between 2,000 and 4,000 m). These estimates of sinking rates are conservative, as these calculations assume that all cells sinking below the photic layer are alive, when available reports indicate that this is not the case^34^. Assuming that only 50% of the cells leaving the photic layer are alive, as observed in the epipelagic samples collected to initiate the decay experiments (mean±s.e.% living cells=48.5±3.8%), the sinking rates for the community to support 18% of living cells at 3,000 m depth ranges from 208 to 732 m d^−1^.

The range of sinking rates of 124–732 m d^−1^ suggested by our experiments and observations of the fraction of living cells in the deep-sea communities is comparable to the sinking rates reported for fast-sinking particles such as aggregates^19^,^21^,^35^ and faecal pellets^12^,^36^,^37^. Moreover, laboratory experiments have demonstrated that diatom cells can remain alive when packaged in faecal pellets^20^. Hence, we infer that the well-preserved phytoplankton cells we observed across the deep-sea must have been injected at depth embedded within fast-sinking aggregates, which may have disaggregated *in situ* or during the retrieval with the Bottle-Net instrument^38^. Hence, our results extend existing evidence of the penetration of fast-sinking particles^19^,^22^ to the deep, oligotrophic ocean.

Our results help constraint the fraction of the microphytoplankton community reaching the deep sea^39^. The pool of phytoplankton cells integrated over the 2,000- to 4,000-m water column represented 0.25% of that present in the photic layer (Table 1). This is a high fraction considering that only between 5 and 10% of microplankton production, which should be lower than the stock on a daily basis, sinks below the mixed layer in the warm waters sampled here^8^,^40^ and that this sinking flux is rapidly attenuated with depth in the oligotrophic ocean^41^,^42^,^43^. For comparison, the fraction of primary production expected with the sedimentary flux at 2,000–4,000 m depth would be 0.04–0.18% (central value 0.08%). The existence of a large, rapid and predictable seasonal pulse of particulate matter to the deep sea in the North Pacific Subtropical Gyre has been reported recently^16^, again suggesting the operation of mechanisms accelerating the export of phytoplankton cells to the deep^35^ sea. Indeed, multiple observations have converged to suggest sinking fluxes from 10 to 1,000 m d^−1^ (refs 11, 15, 22, 36), consistent with the sinking rates of 124–732 m d^−1^ derived here.

The greater dominance of diatoms in the deep-sea microphytoplankton community relative to that in surface waters (Table 1) suggested a role for opal ballasting in delivering the particles containing these cells to the deep sea^44^,^45^. Whereas this observation does not preclude a role for calcite ballasting, coccolitophores (included within the ‘other' microalgal category in Table 1) played a comparatively small role as members of the communities in both surface and deep-sea waters.

Identification in the past of well-preserved phytoplankton cells in the deep sea sampled with 3–5 l bottles was possible because of the phenomenal masses of cells occasionally found, such as 50,000 diatom cells per l at 900 m depth in the Pacific Ocean^23^, which must derive from mass sinking events^16^. However, sampling limitations in the past suggested observations of well-preserved phytoplankton cells in the deep sea to be a rare event^22^,^23^,^24^. Use of the Bottle-Net instrument, capable of quantitatively sampling deep ocean microplankton communities by filtering tens of m^3^ of deep-sea water, and sequencing of 16S rDNA concentrated from hundreds of litres of deep-sea water now demonstrate the presence of well-preserved phytoplankton at depth to be the norm^45^.

Our results conclusively demonstrate that (1) the presence of healthy phytoplankton cells in the deep sea is ubiquitous at the global scale; (2) the abundance of phytoplankton in the deep sea is significant, and these communities show a higher dominance of diatoms relative to those in the upper ocean from which they originate; (3) identify the Equatorial Pacific as an area of high input of phytoplankton cells to the deep sea; and (4) provide evidence that these cells must have reached the bathypelagic ocean down to 4,000 m through fast-sinking mechanisms. Therefore, the results from the global survey presented here confirm the belief that fast-sinking processes should be able to inject fresh organic carbon down to the bathypelagic ocean^45^,^46^,^47^. Phytoplankton cells reaching the deep sea alive will eventually die, as supported by experimentally evidence provided here (Fig. 5) together with the 82% proportion, on average, of dead cells in the deep-sea phytoplankton community. Thus, the ubiquitous rapid transport of fresh phytoplankton cells to the deep ocean helps explain the high bacterial metabolic rates in the deep ocean^18^,^29^,^48^, which are difficult to reconcile if the organic carbon flux was dominated by slowly sinking detritus^45^,^47^,^49^. Rapid sinking of phytoplankton has also been invoked as a likely explanation for the observation of phytodetritus ‘fluff' layers in the sea bed^49^,^50^. Whereas the development of these phytodetritus layers in the sea bed is believed to derive from episodic events^49^,^50^, our results provide evidence that healthy phytoplankton cells also reach the deep ocean outside of such events. Our confirmation that pathways for the rapid delivery of fresh organic carbon by the biological pump typically reach the deep ocean contributes, therefore, to understand the supply of carbon supporting bathypelagic microbial communities.

## Methods

### Malaspina 2010 circumnavigation expedition

The Malaspina 2010 Circumnavigation Expedition sailed the oceans on board R/V Hésperides of the Spanish Navy from 15 December 2010 to 14 July 2011, surveying the Atlantic, Indian and Pacific Oceans^26^,^27^,^28^. The expedition left Spain sailing south via the Atlantic Ocean to enter the Indian Ocean south of Cape Town (South Africa); to enter the Pacific Ocean through the Bass Straight (Australia), and returning to the Northern Hemisphere in May 2011, crossing the Pacific to enter the Atlantic Ocean through the Panama Canal, sailing across the Atlantic to return to Spain. The expedition sampled mostly the subtropical, oligotrophic ocean (Fig. 1), with the sequence of the survey leading to sampling mostly during spring and summer. The expedition sampled the open ocean down to 4,000 m depth, or the maximum depth available if shallower than 4,000 m. Microphytoplankton samples integrated between 4,000 and 2,000 m depth were collected at 58 stations using a Rosette sampling system fitted with a CTD, 12-l Niskin Bottles, and a Bottle-Net sampling device (see below).

### Bottle-Net description

The Bottle-Net (Supplementary Fig. 4) is a new oceanographic device specifically developed for the Malaspina 2010 Circumnavigation Expedition (Pat: ES 2377070 B2), which consists of a conical plankton net housed in a cylindrical PVC pipe that acts as a case, with a net mouth arranged at the top. The case is opened at the bottom but presents a remote-closing cover on the top that hermetically closes the entrance of the net, with a mechanism identical to that of the Niskin bottles. The equipment is designed to be mounted on a standard rosette sampler of oceanographic bottles, where it replaces one 12-l bottle, and uses the same remote shooter as that operating regular oceanographic bottles. The Bottle-Net can also works autonomously when attached to a ballasted oceanographic cable. The Bottle-Net samples only during the ascension of the oceanographic rosette taking one sample per deployment. During sampling, the top cover of Bottle-Net remains opened while the oceanographic rosette descends; however, when the rosette reaches the maximum depth, 4,000 m in our survey, and starts the ascent, the Bottle-Net starts filtering water and collecting organisms. After rising to the target depth, 2,000 m in our survey, the remote shooter is activated and the cover hermetically closes the mouth of the Bottle-Net preventing the entrance of water during the final ascend of the device to the surface. On board, the net must be softly rinsed with filtered seawater in order to retrieve the sample from the collector. Sample volume is estimated as the product between the area of the mouth of the Bottle-Net and the vertical distance covered by the device from the start of the ascension to the closure of the mouth. The Bottle-Net presents an open area ratio of 4, displaying an efficiency of filtration of 96% for deep tows (2,000–4,000 m) and trawling velocities ∼30 m min^−1^ (that is, standard rosette retrieval velocities).

### Bathypelagic microplankton abundance and viability

A fraction of the Bottle-Net sample was stained fresh with the vital stain Back-light Kit (Molecular Probes) to identify living and dying phytoplankton cells. The Bac-light viability Kit (Molecular Probes Invitrogen) is a double-staining technique used to test cell membrane permeability by selective fluorescence signals^51^, proven to be an effective method for phytoplankton^31^,^32^. Living phytoplankton cells with intact membranes fluoresce green (Syto 9, nucleic acid stain) and dead phytoplankton cells with compromised membranes fluoresce red (Propidium Iodine, nucleic acid stain). The samples were filtered on black Nuclepore filters, stained with the Bac-light viability Kit, placed in slides and maintained frozen at −80 °C until examination under epifluorescence microscopy^31^,^32^. The samples were examined under blue light, with those from the Atlantic, Indian and S. Pacific Oceans examined on board the research vessel under a Nikon epifluorescence microscope, and samples from the N. Pacific and the last leg on the Atlantic Ocean (Cartagena de Indias, Colombia, to Cartagena, Spain) examined at the IMEDEA laboratory under a Zeiss Axioplan Imaging and Leica epifluorescence microscopes. The fluorescence of the stained cells is well preserved at −80 °C for several months and samples transported to the laboratory were maintained at −80 °C during the transport.

Blank tows to test possible contamination of the Bottle-Net samples with surface plankton consisted of closing the Bottle-Net at 4,000 m and ascending closed to the surface, and were run in the S. Atlantic, the S. Pacific and the N. Atlantic Oceans, although in the last test the blank was taken from 500 m to surface because of operational reasons. The abundance of phytoplankton found in the blank samples was very small averaging 10.3 cells m^−3^ in the samples collected in the blanks, seven times lower than the abundance found in the parallel Bottle-Net tows at the same stations. The abundance in the blanks is equivalent to 5.97±5.1 × 10^3 ^cells m^−2^ if integrated to the water column sampled, equivalent to the minimum abundance value obtained in the Bottle-Net tows along the study.

Another fraction of the sample collected by the Bottle-Net was fixed with Lugol for further examination at the laboratory. For a comparison with the surface layer, phytoplankton, ciliates and radiolaria were counted in the samples of the Bottle-Net and compared with those in the surface plankton net (200 m) samples (as described below).

Subsamples were sedimented in 50-ml chambers for at least 24 h before enumeration at × 100–200 magnifications using an inverted microscope (AXIOVERT35, Zeiss). For each sample we enumerated microplankton cells at least in half of the sedimentation chamber. Ciliates were identified to the genus level when possible^52^. Radiolaria was quantified as a whole according to Haeckel 53. Surface abundance was always higher than that for samples collected in the Bottle-Net, except for radiolaria in St 143.

### Decay rates of living microphytoplankton cells

The cell mortality rates of phytoplankton living cells in the dark and cold temperature conditions encountered in the bathypelagic ocean were examined with phytoplankton communities collected by vertical tows from the photic layer of the Indian, Pacific and Atlantic Oceans. An aliquot of the photic layer microphytoplankton sample was resuspended in 2 l of 4,000 m water and incubated in the dark at 4 °C for 1–2 months. The community was sampled at the onset of the experiment and at increasing time intervals (1, 2, 3, 4, 7, 14, 20, 30, 40, 60 days), and stained fresh with the vital stain Bac-light Kit, prepared and examined under epifluorescence microscope as described above to quantify the living cells in the community. The half-life (that is, the time for the number of living cells to decline to 50%) and the decay rate of the living cell population were then calculated from the decline in living cells over time.

### Phytoplankton abundance from the surface layer

Samples for micro- and nanophytoplankton enumeration were taken from the Niskin bottles at three levels, surface (3 m), the depth receiving 20% of photosynthetically active radiation (PAR) incident below the surface and the depth of the deep chlorophyll maximum, as established using the PAR and fluorescence sensors fitted in the CTD. Subsamples of 250 ml were introduced in amber glass bottles and fixed with 0.4% hexamine-buffered formaldehyde. For analysis, 100 ml of water were settled in composite chambers during 48 h. Subsequently, two or more transects of the chamber bottom were examined by means of an inverted microscope^54^, under 312 X magnification, to count the smaller cells. Additionally, the whole chamber bottom was scanned under 125 X to enumerate larger, less frequent forms. Organisms were classified to species when possible, but many taxa had to be pooled in categories such as ‘small flagellates' and ‘small dinoflagellates'.

Vertical net hauls were performed between 200 m depth and the surface, with a net of 28-cm mouth diameter and a mesh size of 47 μm. A rough estimate of the water volume filtered by the net was obtained from the expression *πr*^2^*d*, were *r* is the radius of the net and *d* the towing distance (200 m). The filtered plankton was washed into the collecting bucket, which was topped up to a suspension volume of 250 ml. As part of the Malaspina Expedition net phytoplankton collection, a subsample of 140 ml was placed into an amber glass bottle and fixed to a final concentration of 4% hexamine-buffered formaldehyde. For qualitative phytoplankton examination, 2 ml of this subsample were placed into a chamber and examined with an inverted microscope.

### DNA analyses

*Sample collection*: Samples for DNA at 4,000 m depth were collected from 31 stations along the Malaspina 2010 Circumnavigation Expedition and filtered on two size fractions (small 0.2–0.8 μm and large 0.8–20 μm) using 142-mm polycarbonate membrane filters. For each sample 120 l of seawater were first filtered through 200 and 20 μm mesh to remove large plankton. Further filtering was carried out by filtering water serially through 142-mm filters of 0.2 and 0.8 μm pore size with a peristaltic pump (Masterflex, EW-77410-10). Filters were then flash-frozen with N_2_ and stored at −80 °C until DNA extraction.

Filters were cut into small pieces with sterile razor blades and half of every filter was resuspended in 3 ml of lysis buffer (40 mM EDTA, 50 mM Tris-HCl, 0.75 M sucrose). Lysozyme (1 mg ml^−1^ final concentration) was added and samples were incubated at 37 °C for 45 min with slight movement. Then, sodium dodecyl sulfate (1% final concentration) and proteinase K (0.2 mg ml^−1^ final concentration) were added and samples were incubated at 55 °C for 60 min under slight movement. The lysate was collected and processed with the standard phenol–chloroform extraction procedure. An equal volume of Phenol:CHCl_3_:IAA (25:24:1, vol:vol:vol) was added to the lysate, carefully mixed and centrifuged 10 min at 3,000 r.p.m., the aqueous phase was recovered and the procedure was repeated. Finally, an equal volume of CHCl_3_:IAA (24:1, vol:vol) was added to the recovered aqueous phase in order to remove residual phenol. The mixture was centrifuged and the aqueous phase was recovered for further purification. The aqueous phase was then concentrated by centrifugation with a Centricon concentrator (Millipore, Amicon Ultra-4 Centrifugal Filter Unit with Ultracel-100 membrane). Once the aqueous phase was concentrated, this step was repeated three times adding 2 ml of sterile MilliQ water each time in order to purify the DNA. After the third wash, between 100 and 200 μl of purified total genomic DNA product per sample was recovered. Extracted DNA was quantified using a Nanodrop ND-1000 spectrophotometer (NanoDrop Technologies Inc., Wilmington, DE, USA) and the Quant_it dsDNA HS Assay Kit with a Qubit fluorometer (Life Technologies, Paisley, UK).

*Sequencing and sequence data processing*: All library construction and sequencing were carried out at JGI ( www.jgi.doe.gov) following ref. 55. Briefly, the variable region V4 of the 16S rDNA gene was targeted using F515/R806 primers (5′-GTGCCAGCMGCCGCGGTAA-3′/5′-GGACTACHVGGGTWTCTAAT-3′) and sequenced using Illumina MiSeq with 2 × 250 bp reads configuration. All the samples were run in a single lane using unique barcodes for every sample (that is, multiplexing). Before sequencing, a PhiX spike-in shottgun library reads were added to the amplicon pool for a final concentration of ∼20–25% of the pair-end reads library.

Reads were first scanned for PhiX reads and contaminants (for example, Illumina adapter sequences) and all disrupted pair-end reads (every read pair for which one read has been lost because of the screening) were discarded. The remaining reads were trimmed to 165 bp and assembled using the FLASH software^56^, and primer sequences were removed from the assembled reads. Assembled reads were trimmed from both 5′ and 3′ ends using a 20-bp sliding window (mean quality threshold >30). Trimmed reads with more than 5 or 10 nucleotides below quality 15 were discarded. Clustering was carried out using an in-house algorithm at JGI that consisted of clustering the filtered reads using USEARCH at 99% identity and clusters having abundances lower than 3 reads were discarded. A final cluster at 97% identity of the remaining clusters was carried out and the clusters obtained were considered as operational taxonomic units (OTUs). Finally, the obtained OTUs were checked for chimeric sequences using both the Chimera Slayer algorithm as implemented in the MOTHUR software^57^ and the UCHIME reference-based algorithms^58^. OTUs detected as chimeric sequences by any of these methods were removed. Non-chimeric OTUs were taxonomically annotated using both the online RDP Naive Bayesan Classifier^59^ and the BLAST-based classifier within the QIIME pipeline^60^ using the SILVA database (release 108) as reference.

A total of 8,141,076 raw reads were obtained for the whole data set from which 42,254 reads corresponded to contaminant reads (that is, Illumina adapter sequences) and 1,526,330 to PhiX reads. That left a total of 6,572,492 non-contaminant non-PhiX reads from which 3,100,410 reads could be paired and assembled. From these, 2,584,926 reads passed the filtering process described before.

We considered all the sequences that were annotated by the two algorithms as belonging to cyanobacteria, and those annotated as belonging to algal chloroplasts, and expressed them as a percentage of the total recovered sequences. In the few cases in which the SILVA taxonomy did not agree with the RDP taxonomy at these large level, we followed the SILVA annotation.

### Flow cytometry

The abundance of pigment-containing prokaryotes in deep ocean samples were obtained using flow cytometry analyses of DNA-stained samples. Samples of 2 ml of seawater sampled from 4,000 m depth were fixed with 1% paraformaldehyde and 0.05% glutaraldehyde (immediately after collection, and after 15 min at room temperature in the dark they were deep-frozen in liquid nitrogen. A few days after sampling they were unfrozen, stained with SybrGreen I (Molecular Probes, Invitrogen) at a 1/10,000 dilution and run in a BectonDickinson FACSCalibur flow cytometer equipped with a blue 488-nm 15-mW Argon-ion laser as explained elsewhere^61^. At least 100,000 events were recorded and photosynthetic prokaryotes could be distinguished in a DNA-derived green fluorescence against a chlorophyll-derived red fluorescence scatter plot. Calibration of the machine for absolute counts was made daily by measuring the exact volume being analysed. Fluorescent beads (1 μm, Fluoresbrite carboxylate microspheres, Polysciences Inc., Warrington, PA) were added at a known density as internal standards.

## Additional information

**How to cite this article:** Agusti, S. *et al.* Ubiquitous healthy diatoms in the deep sea confirm deep carbon injection by the biological pump. *Nat. Commun.* 6:7608 doi: 10.1038/ncomms8608 (2015).

## Supplementary Material

Supplementary Information Supplementary Figures 1-4 and Supplementary Table 1

## Figures and Tables

**Table 1:** Mean (±s.e.) absolute and relative abundance of phototrophic and heterotrophic plankton in the surface and deep ocean.

	**Cell abundance (cells m**^**−2**^)	**s.e.**	***N***
*Surface ocean (0–200 m)*			
Microphytoplankton	9.78 × 10^7^	3.63 × 10^7^	11
Diatoms	6.73 × 10^7^	3.08 × 10^7^	11
Dinoflagellates	3.01 × 10^7^	8.88 × 10^6^	11
Others	7.61 × 10^5^	4.49 × 10^6^	11
Cyanobacteria	1.29 × 10^6^	1.26 × 10^6^	11
Ratio Diatoms/Dinoflag	2.5	0.9	11
Ciliates	9.15 × 10^6^	2.83 × 10^6^	11
Ratio Phytop/Ciliates	9.9	2.4	11
Picophytoplankton	2.72 × 10^13^	1.10 × 10^12^	226
Heterotrophic bacteria	1.33 × 10^14^	1.24 × 10^13^	123
Ratio Bact/Picophytop	4.9	11.3	123
			
*Deep ocean (2,000–4,000 m)*			
Microphytoplankton	2.52 × 10^5^	5.29 × 10^4^	58
Diatoms	2.02 × 10^5^	5.08 × 10^4^	58
Dinoflagellates	3.46 × 10^4^	5.61 × 10^3^	58
Others	1.12 × 10^3^	3.04 × 10^3^	58
Cyanobacteria	5.71 × −10^2^	4.21 × 10^1^	58
Ratio Diatoms/Dinoflag	13.9	3.5	58
Ciliates	9.95 × 10^4^	2.24 × 10^4^	11
Ratio Phytop/Ciliates	8.9	2.9	11
Picophytoplankton	3.16 × 10^12^	1.05 × 10^3^	123
Heterotrophic bacteria	1.20 × 10^14^	2.62 × 10^14^	123
Ratio Bact/Picophytop	38.0	24.9	123

Bact/Picophytop, heterotrophic bacteria to picophytoplankton cell abundance ratio; Cyanobacteria, filamentous forms; Diatoms/Dinoflag, diatoms to dinoflagellates cell abundance ratio; N, number of samples; Others, flagellates and silicoflagellates; Phytop/Ciliates, phytoplankton to cilliates cell abundance ratio..
