# Supplementary material for: Ubiquitous healthy diatoms in the deep sea confirm deep carbon injection by the biological pump
Source: Nat Commun. 2015 Jul 9;6:7608. doi: 10.1038/ncomms8608 (PMC4510647; doi:10.1038/ncomms8608)
Supplement: Supplementary Information — Supplementary Figures 1-4 and Supplementary Table 1 [file ncomms8608-s1.pdf]

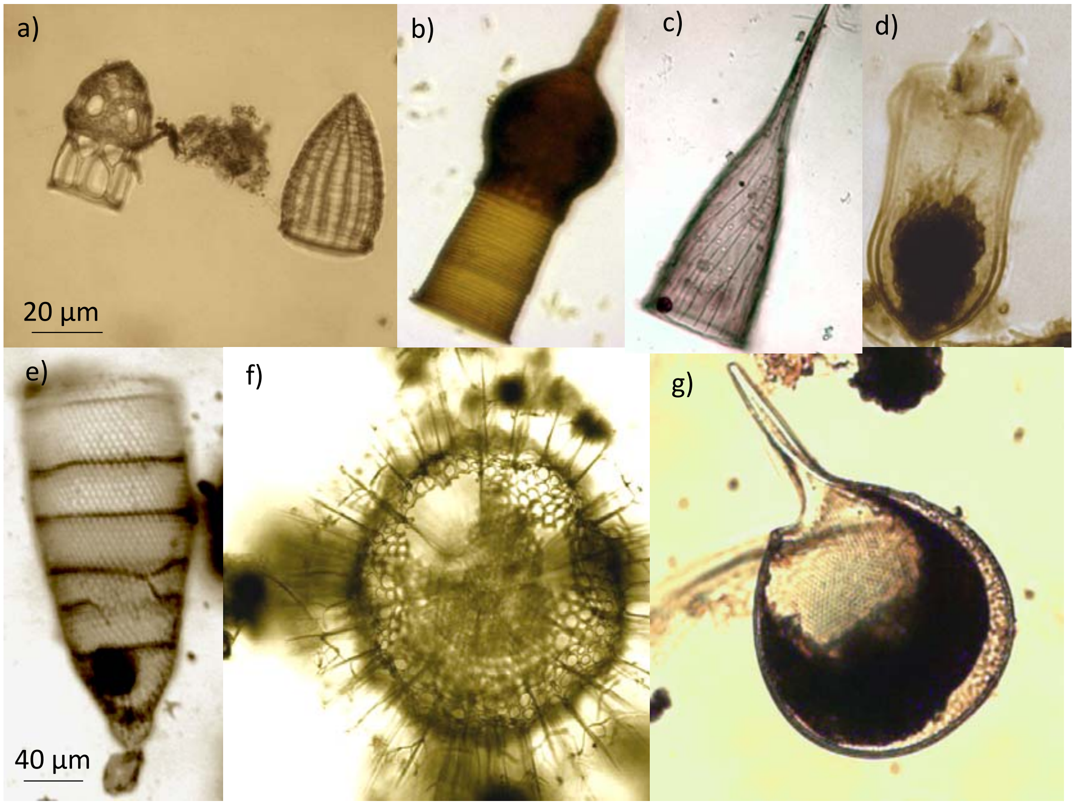

Supplementary Figure 1**.** Tintinnid ciliates (A, B, C, D) and radiolaria (E, F, G) collected by the bottle net between 2,000-4,000 m.

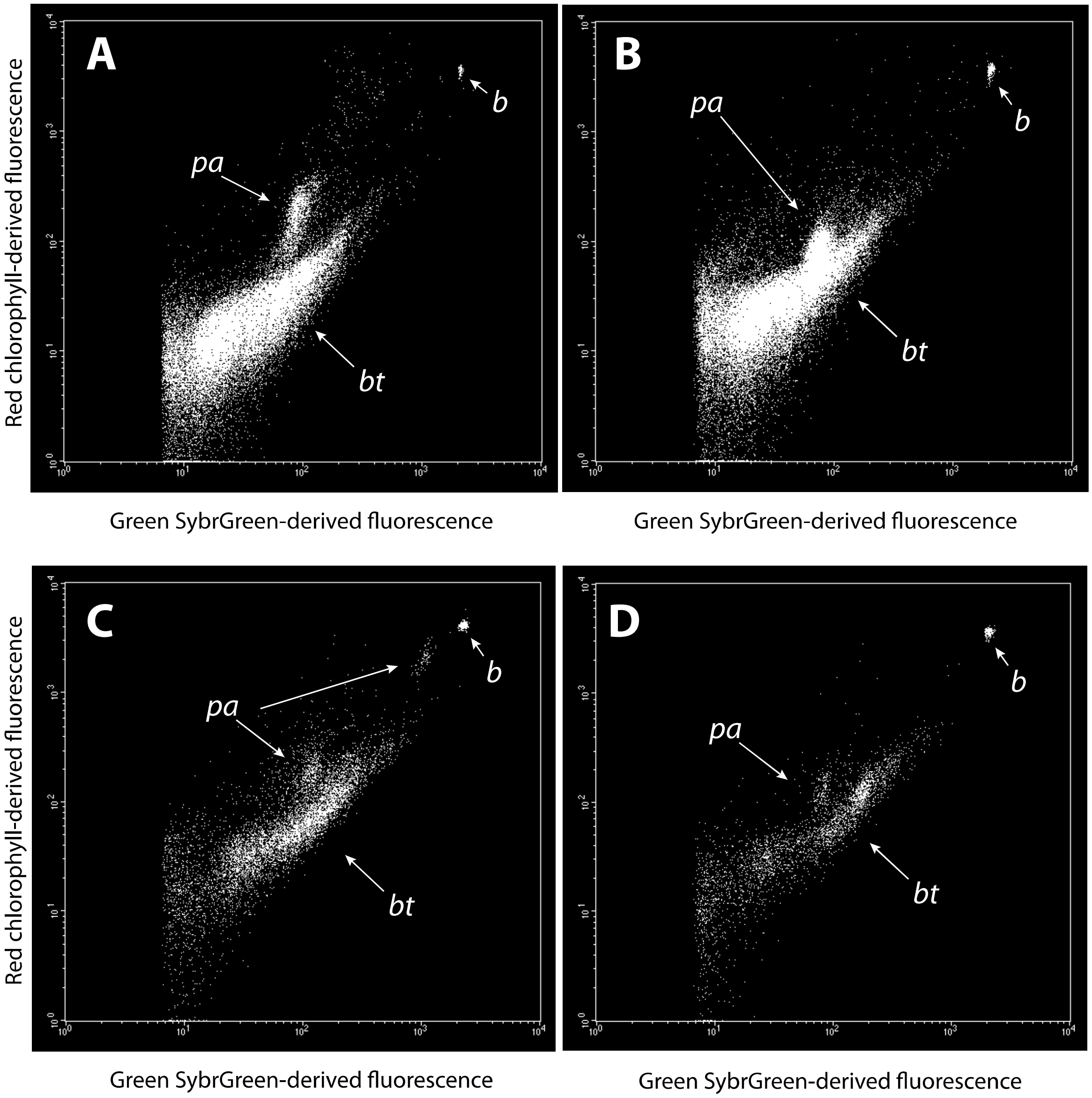

Supplementary Figure 2**.** Cytograms of some selected surface and deep ocean samples. The samples were stained with SybrGreen I, a DNA stain that targets nucleic acids and, thus, stain all microbes, phototroph or autotroph. However, those microbes that have red autofluorescence from the chlorophyll a, appear in a different diagonal when plotting red vs. green (SybrGreen) fluorescence. They are indicated as "pa", while the bacteria and archaea are labelled as "bt". Reference 1 µm Yellow-Green Polysciences beads were added as internal standards (labelled "b"). A) A surface sample, Station 40 at 70 m, ratio bt/pa= 11.8; B) Station 110, at 2000 m, ratio bt/pa= 6.1; C) Station 126, at 2200 m ratio bt/pa= 6.2; and D) Stn 113, at 3850 m, ratio bt/pa= 9.1.

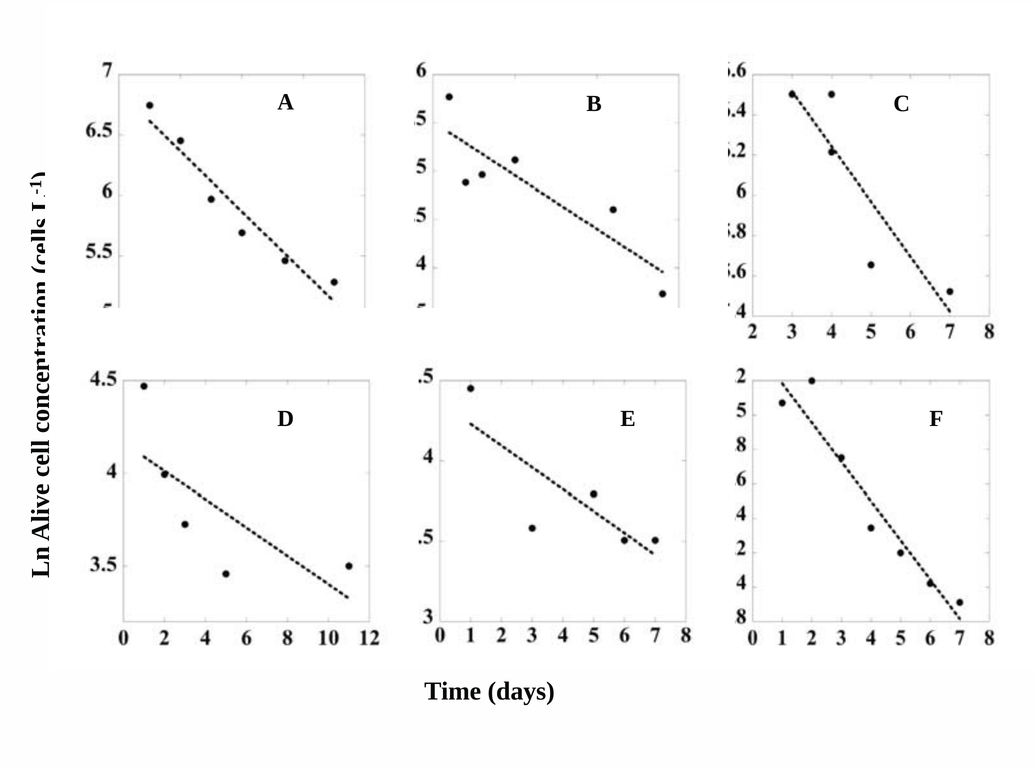

Supplementary Figure 3. Mortality of surface phytoplankton cells in the dark. The decline in the number of alive cells of phytoplankton sampled at the surface layer declined with time when maintained in the dark and at cold temperature, conditions encountered during their possible sinking transient from the surface to the deep ocean. (A) *Trichodesmium* sp. ($p < 0.001$); (B) centric diatom ($p < 0.05$); (C) *Ceratium* sp. ($p < 0.01$); (D) *Ceratium* spp. ($p = 0.07$); (E) *Gymnodinium* sp. ($p = 0.09$); (F) Dinoflagellate ($p < 0.001$).

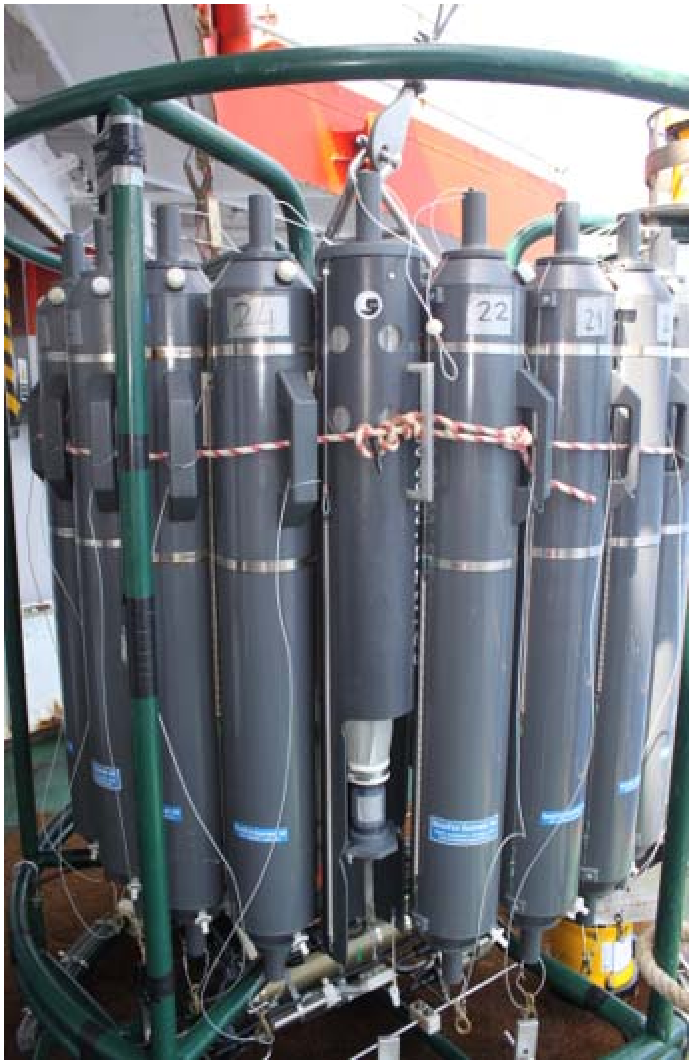

Supplementary Figure 4**.** The new device Bottle-Net installed in the rossette sampler system amid the Niskin bottles.

Supplementary Table 1. Taxonomic composition of the micro-phytoplankton communities found at the surface (0-200 m depth) and deep (2000-4000 m depth) ocean. The stations were selected to shown the variability found at the ocean surface and deep waters in communities across the deep ocean. The cells counted were intact. The symbols are scaled to the relative abundance of the taxon at each station: (x) low abundance, (X) abundant, (XX) dominant, (-) not detected. Pennate diatoms: include *Cylindrotheca* and other genera. Centric diatoms: include a variety of centric genera. *Protoperidinium*, *Gonyaulax*, others: Included cells of those genera and other dinoflagellate genera close morphologically. *Richelia* was found intracellular in *Rhizosolenia*.

Station number	42		47		68	
Latitude (N+,S-)	-32.234		-34.44		-31.554	
Longitude (E+,W-)	9.345166667		31.112		113.467	
	**Surface**	**Deep**	**Surface**	**Deep**	**Surface**	**Deep**
Dinoflagellates						
Ceratium fusus	X	X	XX	X	-	-
Ceratium spp.	X	X	-	-	X	X
Dinophysis spp.	-	-	-	-	-	-
Prorocentrum spp.	x	-	-	-	-	-
Protoperidinium, *Gonyaulax*, others	-	x	-	-	x	x
Diatoms & Dictyochales						
Gymnodinium spp.	X	X	-	-	x	-
Pennate diatoms	X	X	-	-	X	X
Chaetoceros spp., *Bacteriastrum* spp.	-	-	-	-	x	x
Planktoniella sol	-	-	X	-		
Coscinodiscus spp.	-	-	X	X		
Centric diatoms	X	X	-	-		
Rhizosolenia spp.	-	-	X	X	X	X
Hemiaulus spp.	-	-	-	-		
Dictyocha spp.	-	-	x	-		
Cyanobacteria						
Richelia sp.	-	-	-	-	x	x
Trichodesmium spp.	-	-	X	X	-	-

Table 1. Continued

Station number	74		89		94	
Latitude	-39.243		-9.474		1.599	
Longitude	135.139		-172.298		-166.85	
	**Surface**	**Deep**	**Surface**	**Deep**	**Surface**	**Deep**
Dinoflagellates						
Ceratium fusus	-	-	x	-	-	-
Ceratium spp.	X	X	X	X	x	x
Dinophysis spp.	-	-	x	-	-	-
Prorocentrum spp.	x	-	-	-	-	-
Protoperidinium, *Gonyaulax*, others	-	-	x	x	-	x
Gymnodinium spp.	-	-	-	-	X	-
Diatoms & Dictyochales						
Pennate diatoms	-	-	-	-	XX	XX
Chaetoceros spp., *Bacteriastrum* spp.	-	-	-	-	x	-
Planktoniella sol	-	-	x	x	-	-
Coscinodiscus spp.	-	-	-	-	-	-
Centric diatoms	x	x	-	-	x	x
Rhizosolenia spp.	-	-	X	x	X	X
Hemiaulus spp.	-	-	-	-	-	-
Dictyocha spp.	-	-	X	x	-	-
Cyanobacteria						
Richelia sp.	-	-	-	-	-	-
Trichodesmium spp.	-	-	-	-	-	-

Table 1. Continued

Station number	116		129		133	
Latitude	13.205		15.069		18.996	
Longitude	-113.256		-69.295		-55.15	
	**Surface**	**Deep**	**Surface**	**Deep**	**Surface**	**Deep**
Dinoflagellates						
Ceratium fusus	-	-	x	-	x	x
Ceratium spp.	x	x	x	-	X	X
Dinophysis spp.	-	-	-	-	x	-
Prorocentrum spp.	x	x	-	-	-	-
Protoperidinium, *Gonyaulax*, others	-	-	x	x	x	-
Gymnodinium spp.	x	-	-	-	-	-
Diatoms & Dictyochales						
Pennate diatoms	X	X	x	x	-	-
Chaetoceros spp., *Bacteriastrum* spp.	XX	XX	-	-	-	-
Planktoniella sol	x	-	-	-	-	-
Coscinodiscus spp.	-	-	-	-	-	-
Centric diatoms	-	-	x	x	-	x
Rhizosolenia spp.	-	-	-	x	x	-
Hemiaulus spp.	-	-	x	-	x	-
Dictyocha spp.	-	-	-	-	-	-
Cyanobacteria						
Richelia spp.	-	-	-	-	-	-
Trichodesmium spp.	-	-	XX	XX	X	X
